# LINC02257, an Enhancer RNA of Prognostic Value in Colon Adenocarcinoma, Correlates With Multi-Omics Immunotherapy-Related Analysis in 33 Cancers

**DOI:** 10.3389/fmolb.2021.646786

**Published:** 2021-04-30

**Authors:** Junbo Xiao, Yajun Liu, Jun Yi, Xiaowei Liu

**Affiliations:** Department of Gastroenterology, Xiangya Hospital, Central South University, Changsha, China

**Keywords:** LINC02257, enhancer, pan-cancers, immune-related multi-omics analysis, bioinformatic analysis

## Abstract

Accumulated evidence supports that long non-coding RNAs (lncRNAs) are involved significantly in the development of human cancers. Enhancer RNAs (eRNAs), a subtype of lncRNAs, have recently attracted much attention about their roles in carcinogenesis. Colon adenocarcinoma is one of the most commonly diagnosed tumors with unfavorable prognosis. It highlights the great significance of screening and identifying novel biomarkers. More importantly, it remains to be elucidated with respect to the function of eRNAs in colon adenocarcinoma, as is in pan-cancers. The expression of LINC02257 was determined based on the data obtained from The Cancer Genome Atlas (TCGA). Further evaluation was performed on the basis of the following analyses: clinicopathology and survival analysis, gene ontology (GO) terms, and Kyoto Encyclopedia of Genes and Genomes (KEGG) pathway analysis, as well as multi-omics immunotherapy-related analysis and co-expression analysis. The statistical analysis was conducted in R software, and immune cell infiltration of LINC02257 expression in cancers was investigated by using the CIBERSORT algorithm. By large-scale data mining, our study highlighted that a total of 39 eRNA genes were associated with colon adenocarcinoma prognosis, among which 25 eRNAs showed significant associations with their predicted target genes. LINC02257 was identified as the most significant survival-associated eRNA, with DUSP10 as its target gene. Besides, the high expression of LINC02257 in colon adenocarcinoma was more vulnerable to unfavorable prognosis and correlated with various clinical characteristics. GO and KEGG analyses revealed that LINC02257 was closely correlated with extracellular matrix organization via the PI3K-Akt signaling pathway. Besides, LINC02257 expression correlated with a multi-omics analysis of 33 cancer types, such as survival analysis [overall survival (OS), disease-specific survival (DSS), disease-free interval (DFI), and progression-free interval (PFI)] and immunotherapy-related analysis [tumor microenvironment (TME), tumor mutational burden (TMB), and microsatellite instability (MSI)]. Finally, we investigated the co-expression genes of LINC02257 and its potential signaling pathways across different cancer types. LINC02257 is screened and can function as an independent prognostic biomarker through the PI3K-Akt signaling pathway for colon adenocarcinoma. Simultaneously, LINC02257 may be a multifaceted and significant immunotherapy-related eRNA in different cancers.

## Introduction

Colorectal cancer (CRC) encompasses cancers of the colon and the rectum, which has been recognized to be one of the most commonly diagnosed gastroenterological malignancies. It accounts for the second and third leading cause of cancer-related mortality among males and females worldwide, respectively (Esmaeili et al., 2020; Harada and Morlote, 2020). Colon adenocarcinoma is a malignancy that originates from the intestinal epithelium and has a rapid increase in its incidence. Despite remarkable advances in its diagnosis and treatment during the past decades, the prognosis is still not satisfying, with a 5-year survival rate of less than 60% (Zhou et al., 2020). Besides, accumulated evidence supports that frequent postoperative sporadic relapse and/or metastatic recurrence exerts crucial effects on the prognosis of colon adenocarcinoma patients. Accordingly, it is essential to clarify the molecular mechanisms concerning colon carcinogenesis and to explore promising diagnostic and therapeutic biomarkers to improve patients’ prognosis (Vasaikar et al., 2019).

Enhancers are typically considered as regulatory DNA elements that enhance target genes’ transcription, which is bound by RNA polymerase II (RNAP II), transcription factors, and co-regulators. Recently, enhancers have been reported to transcribe non-coding RNAs (ncRNAs), known as enhancer RNAs (eRNAs) (Zhang et al., 2019), large numbers of which have been identified to play pivotal roles in mediating the activation of target genes at the transcription level. Meanwhile, the activation and generation of eRNAs are indispensable in human cancers, specifically in the activation of oncogenes or oncogenic signaling pathways (Kim et al., 2010; Li et al., 2016; Zhang et al., 2019). Previous studies have demonstrated a possible dual effect of eRNAs in the tumorigenesis of cancers. For example, in prostate cancer, Kallikrein-related peptidase 3 eRNA (KLK3e), an eRNA produced by KLK3’s upstream enhancers, can selectively enhance the androgen receptor (AR)-dependent gene expression, resulting in a positive effect on prostate cancer cell proliferation (Hsieh et al., 2014). However, p53-induced eRNAs are required for p53 transcription enhancement and p53-dependent cell-cycle arrest (Melo et al., 2013). Taken together, eRNAs may play crucial roles in tumorigenesis and show the promising clinical prospect of eRNA-targeted therapy (Leveille et al., 2015).

In addition to the development of chemotherapy and radiotherapy, immune-related mechanisms and immunotherapeutic strategies are currently under extensive investigation. Immunotherapy has undergone a tremendous transformation from mechanistically complicated protocols to the forefront of armamentarium for some malignant tumors (Gravitz, 2013), which targets components of tumor microenvironment (TME) or immunotherapy biomarkers of microsatellite instability (MSI) and tumor mutational burden (TMB), specifically in colon adenocarcinoma (Frankel et al., 2017; Chang et al., 2018; Locy et al., 2018; Chan et al., 2019). To the best of our knowledge, there are so far no reports on immunotherapy-associated eRNAs, as well as their underlying functions and mechanisms.

Here, the present study was carried out to identify potential prognostic eRNA, LINC02257, and its target gene in colon adenocarcinoma. Further comprehensive analysis was conducted focusing on LINC02257 expression and association with survival in colon adenocarcinoma patients (Gu et al., 2019; Sun and Ling, 2019). Meanwhile, gene ontology (GO) and Kyoto Encyclopedia of Genes and Genomes (KEGG) analyses were also performed in our subsequent investigation. Moreover, the connection between LINC02257 and 33 cancer types was analyzed by using immunotherapy-related analysis, including TMB, MSI, and TME. It is expected to shed light on the understanding of eRNAs in immune-related treatment among various cancers.

## Materials and Methods

### The Cancer Genome Atlas Data Analysis

The expression of eRNAs in a variety of cancer (Workflow Type: HTSeq-FPKM) was obtained from The Cancer Genome Atlas (TCGA) database^1^, together with related clinical and survival information (Subramanian et al., 2005; Wang et al., 2016). Many collected samples were screened, with data showing insufficient information (age, tumor stage, survival, etc.) excluded. Our study was conducted in line with TCGA publication guidelines^2^. Potential eRNAs with significant correlations with both overall survival (OS) (*p* < 0.05, a false discovery rate (FDR)-adjusted *p*-value < 0.05) and levels of their target genes (*R* > 0.4, *p* < 0.001, FDR-adjusted *p*-value < 0.05) were considered as key eRNAs in colon adenocarcinoma (Gu et al., 2019).

### Gene Set Enrichment Analysis

Gene set enrichment analysis (GSEA) was performed by using normalized RNA-Seq data from TCGA database (Subramanian et al., 2005). GO terms and KEGG pathways were further analyzed to investigate possible biological functions of LINC02257. Specifically, the GO analysis revealed the LINC02257 function in the biology process, cell component, and molecular function, while the KEGG analysis showed the pathway enrichment of LINC02257. To be identified as statistically significant, enrichment results shall meet the following two criteria: a false discovery rate (FDR) < 0.050 and a nominal *p*-value < 0.050.

### Analysis of Immune Cell Infiltrates

Calculation of the stromal and immune scores by using the Estimation of stromal and Immune cells in Malignant Tumors using Expression data (ESTIMATE) algorithm regarding the downloaded RNA expression data and cancer samples was categorized by the median of immune/stromal scores into high- and low-score groups. ESTIMATE output stromal and immune scores performed a single-sample GSEA (Verhaak et al., 2010; Yoshihara et al., 2013; Aran et al., 2015). Furthermore, an algorithm called CIBERSORT^3^ was used for assessing the gene expression among sets in the samples (Newman et al., 2015), with the aim to measure the immune response of 22 tumor-infiltrating immune cells (TIICs), so as to evaluate their association with LINC02257 expression in different types of cancer and to uncover their relationships with TIICs.

### Comprehensive Analysis

Samples from TCGA database were used to analyze the correlation between LINC02257 expression and clinicopathological features of patients with colon adenocarcinoma. Meanwhile, a swarmplot using cancer types as a variable was utilized to illustrate the differential expression of LINC02257. In addition, the OS, disease-specific survival (DSS), disease-free interval (DFI), and progression-free interval (PFI) analysis were performed to analyze the relationship between LINC02257 expression and patients’ prognosis (Uhlen et al., 2015).

### Statistical Analysis

All statistical analyses were conducted using R software (version 3.5.3). The comparison between tumor and normal tissues was evaluated by the non-parametric Wilcoxon signed-rank test. The correlation was assessed by using Spearman’s rank correlation coefficient. Furthermore, both the univariate and multivariate models of the Cox analysis were applied to calculate the 95% confidence interval (CI) and hazard ratio (HR). Univariate survival analysis was used to compare several clinical characteristics with survival rate. Multivariate Cox analysis was further performed to evaluate the effect of LINC02257 expression, along with other pathological and clinical factors (age, gender, tumor size, distant metastasis, and tumor stage) on the OS of patients. R package “mediation” (version 3.5.3) was performed for mediation analysis for age as mediation between LINC02257 and survival. A *p*-value < 0.05 and FDR-adjusted *p*-value < 0.05 was considered to predict the presence of statistically significant difference.

## Results

### Enhancer RNAs of Prognostic Value in Colon Cancer

**FIGURE 1:**
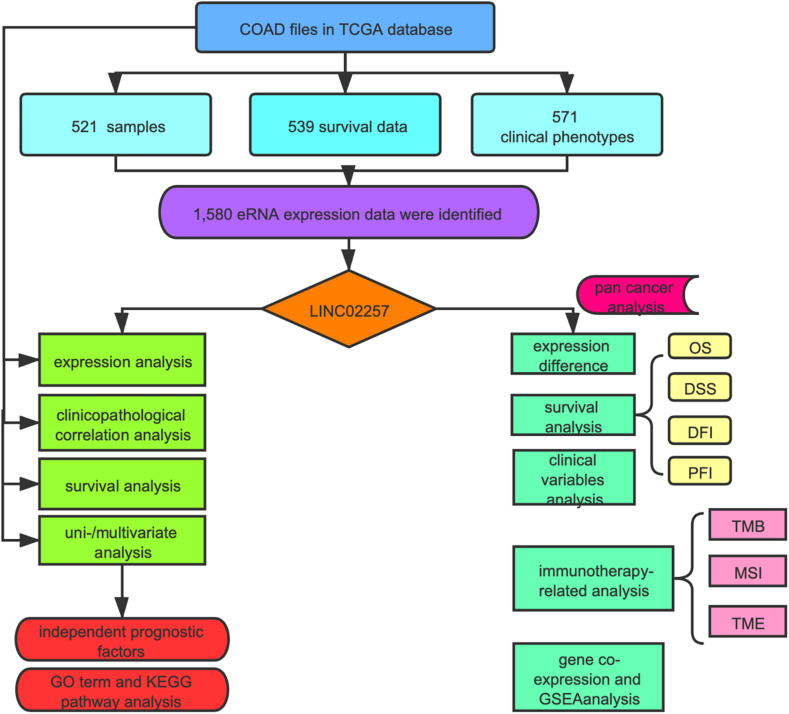
Flowchart of the study.

As indicated in Figure 1, data mining using TCGA database was achieved from a total of 60,484 RNA-seq gene expression data of colon adenocarcinoma samples (*n* = 521), survival data (*n* = 539), and clinical phenotypes (*n* = 571). After that, a targeted number of 1,580 eRNA expression data were extracted from those samples. For an in-depth analysis, the intersection between the screened eRNA expression and survival data was performed with the collection of 1,570 eRNA expression data of 449 samples. Two subgroups of high and low expression groups were divided based on the median value of eRNA expression, and 39 eRNA gene expressions were associated with the prognosis of colon adenocarcinoma (Supplementary Table 1, Kaplan–Meier log-rank test, *p* < 0.05, FDR-adjusted *p*-value < 0.05). In addition, significant correlation levels of these 39 eRNAs with their predicted target genes were only in 25 eRNAs (Spearman’s rank correlation coefficient *r* > 0.4, *p* < 0.001, FDR-adjusted *p*-value < 0.05; Table 1). Specifically, LINC02257 and its predicted target DUSP10 (Png et al., 2016) were identified as the most prognostic eRNA with the least p-value of Kaplan–Meier log-rank test regarding the previous 25 eRNAs.

**TABLE 1:** List of overall survival associated genes derived from enhancers.

**eRNA**	**KM**	**Target**	**cor**	**corPval**	**FDR-adjusted *p*-value**
AC012368.1	0.04831255	PELI1	0.32603608	3.98E−13	4.01E−13
AC022784.1	0.00500228	PPP1R3B	0.13996317	0.00233081	0.00117483
AC046134.2	0.03415434	RBP1	0.10316519	0.02519561	0.01147073
AC053527.2	0.00236009	ALB	0.16513588	0.00031934	0.00017334
AC092944.1	0.01159129	CCNL1	0.33978972	3.42E−14	3.71E−14
AC108751.4	0.02061273	TM4SF1	0.35391784	2.41E−15	3.09E−15
AC108751.4	0.02061273	TM4SF18	0.07701974	0.09500592	0.03943662
AC139149.1	0.04345036	BAHCC1	0.25244199	2.79E−08	2.07E−08
AL139246.2	0.04486095	TNFRSF14	0.2854273	2.79E−10	2.32E−10
AL353747.3	0.03940883	UNC93A	0.57486828	8.86E−43	4.17E−42
AL683813.2	0.01629478	ARHGEF6	0.14910866	0.00117204	0.00061264
AP003555.1	0.00550634	ANO1	0.58781578	4.19E−45	2.95E−44
AP003555.2	0.00450645	ANO1	0.50814036	2.75E−32	9.69E−32
APELA	0.04674045	TRIM61	0.30716899	9.49E−12	8.37E−12
C2orf92	0.02682689	COX5B	−0.1376607	0.00277498	0.00135048
ELFN1	0.00517534	MAFK	0.34260487	2.74E−14	3.22E−14
ELFN1	0.00517534	TMEM184A	0.18242344	7.02E−05	4.31E−05
ELFN1	0.00517534	PSMG3	0.1089876	0.01801643	0.00847569
ELFN1-AS1	0.00372687	TMEM184A	0.31592828	2.94E−12	2.76E−12
LINC00174	0.03760549	TPST1	−0.0886271	0.05461454	0.02408719
LINC00649	0.00900198	SLC5A3	0.21018374	4.21E−06	2.83E−06
LINC00649	0.00900198	MRPS6	0.08409923	0.06822039	0.02917616
LINC02257	0.00012532	DUSP10	0.43606423	2.78E−23	5.60E−23
LINC02381	0.04837723	HOXC4	0.61105361	1.50E−49	2.11E−48
LINC02381	0.04837723	HOXC8	0.47911944	2.11E−28	5.94E−28
LINC02381	0.04837723	HOXC6	0.45614791	1.40E−25	3.29E−25
LINC02381	0.04837723	HOTAIR	0.20178279	1.02E−05	6.54E−06
LINC02381	0.04837723	HOXC11	0.17679999	0.00011467	6.74E−05
LINC02381	0.04837723	HOXC13	0.16609247	0.00029435	0.00016617
MIR4435-2HG	0.04207077	BCL2L11	0.2362846	2.31E−07	1.63E−07
MYOSLID	0.01159681	KLF7	0.27154907	2.09E−09	1.64E−09
STEAP1B	0.01517609	IL6	0.40453792	5.68E−20	8.91E−20
TMEM184A	0.02072743	MICALL2	0.37560806	2.69E−18	3.80E−18
ZNRF2P2	0.03039512	PRR15	0.40759279	2.81E−20	4.96E−20

*Spearman’s rank correlation coefficient *r* > 0.4, *p* < 0.001. FDR-adjusted *p*-value < 0.05 was considered statistically significant. eRNA, enhancer RNA; FDR, false discovery rate.*

### High Expression of LINC02257 Correlates With Clinicopathological Variables and Predicts Unfavorable Prognosis in Colon Adenocarcinoma

**FIGURE 2:**
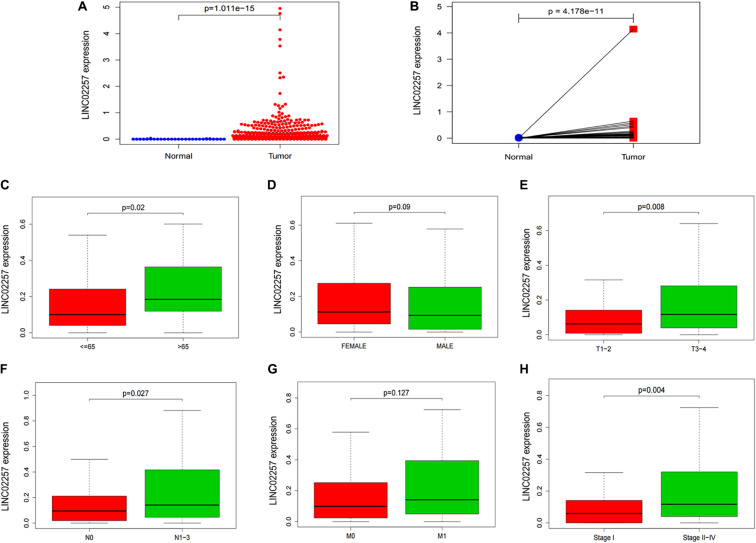
LINC02257 is correlated with multiple Clinicopathological variables in COAD **(A)** LINC02257 expression in 39 normal tissues and 398 tumor tissues via Wilcox test in a swarm plot, **(B)** LINC02257 expression between the paired tumor tissues and the normal tissues in the same sample. **(C–H)** High expression of LINC02257 is related to the patients age, T, N and stages I–IV and shows no significant correlation with gender and M (*p* < 0.05 was considered significant).

**FIGURE 3:**
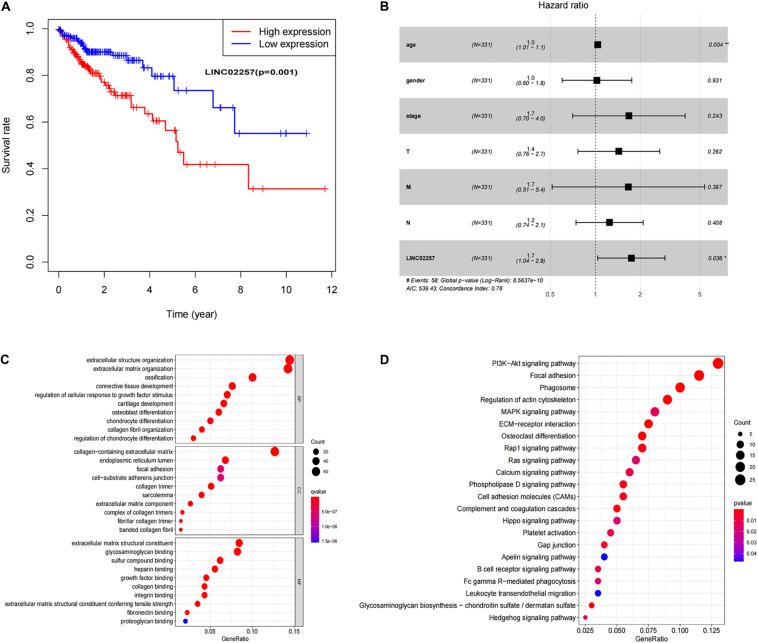
Survival analysis of LINC02257 and GO term and KEGG pathway analysis in COAD **(A)** LINC02257 related to the survival time of patients, and with the increase of LINC02257 expression, the prognosis of colon cancer patients was worse. **(B)** Multivariate COX regression analysis suggests that age and expression of LINC02257 could be independent factors for the prognosis survival of colon cancer patients. **(C)** GO enrichment analysis shows that the biological processes and molecular functions are strongly associated with LINC02257 in extracellular structure organization, ossification, collagen fibril organization and connective tissue development. **(D)** KEGG pathway analysis shows five pathways of the significant correlation with LINC02257 expression: PI3K-Akt signaling pathway. Focal adhesion, Phagosome, Regulation of actin cytoskeleton and MAPK signaling pathway (*p* < 0.05 was considered significant).

Data extraction was performed concerning the expression of LINC02257 in tumor and normal tissue samples from colon adenocarcinoma in TCGA database. Wilcoxon test was used to identify the difference of LINC02257 expression in a total of 398 tumor files and 39 normal files plotted on a swarmplot (*p* < 0.05, Figure 2A). Additionally, there was significant difference in LINC02257 expression between the paired tumor tissues and the normal tissues in the same sample (*p* < 0.05, Figure 2B). Furthermore, according to the correlation analysis between LINC02257 and a variety of clinicopathological variables, the increased expression of LINC02257 was related to patient age (*p*-value = 0.02, Figure 2C), T stage (*p*-value = 0.008, Figure 2E), N stage (*p*-value = 0.027, Figure 2F), and stages I–IV (*p*-value = 0.004, Figure 2H). Besides gender (*p*-value = 0.19, Figure 2D) and M stage (*p*-value = 0.127, Figure 2G) show no significant correlation with LINC02257 expression. In accordance with the Cox analysis on the relationship between LINC02257 expression and OS, it was found that the expression of LINC02257 was related to the survival time of patients, and a worse prognosis was found along with the increase of LINC02257 expression (Kaplan–Meier log-rank test, *p* = 0.001, Figure 3A). As illustrated in Table 2, some parameters, revealed by a univariate analysis of correlation, such as age (HR = 1.030, *p*-value < 0.018, FDR-adjusted *p*-value < 0.036), stage (HR = 2.502, *p*-value < 2.48E-09, FDR-adjusted *p*-value < 1.49E-08), T stage (HR = 2.927, *p*-value < 4.97E−05, FDR-adjusted *p*-value < 1.99E-04), N stage (HR = 5.226, *p*-value < 1.25E−09, FDR-adjusted *p*-value < 2.20E-06), M stage (HR = 2.175, *p*-value < 4.41E−07, FDR-adjusted *p*-value < 8.78E-09), and LINC02257 expression (HR = 1.911, *p*-value < 0.003, FDR-adjusted *p*-value < 0.009) were obviously associated with OS. Multivariate analysis, as shown in a forest boxplot in Figure 3B, revealed that age (HR = 1.037, *p*-value < 0.004) and LINC02257 expression (HR = 1.738, *p*-value < 0.036) were independent prognostic factors for colon adenocarcinoma patients.

**TABLE 2:** Univariate analysis and multivariate analysis of the correlation of LINC02257 expression with OS among colon adenocarcinoma patients.

**Parameter**	**Univariate analysis**					**Multivariate analysis**			
	**HR**	**HR.95L**	**HR.95H**	***p***	**FDR-adjusted *p*-value**	**HR**	**HR.95L**	**HR.95H**	***p*-value**
Age	1.030	1.005	1.055	**0.018**	**0.036**	1.037	1.012	1.063	**0.004**
Gender	1.126	0.668	1.899	0.655	0.655	1.024	0.599	1.751	0.930
Stage	2.502	1.851	3.382	**2.48E−09**	**1.49E−08**	1.675	0.705	3.980	0.243
T classification	2.927	1.742	4.917	**4.97E−05**	**1.99E−04**	1.434	0.764	2.695	0.262
N classification	5.226	3.065	8.911	**1.25E−09**	**2.20E−06**	1.661	0.513	5.375	0.397
M classification	2.175	1.609	2.941	**4.41E−07**	**8.78E−09**	1.243	0.742	2.086	0.408
LINC02257	1.911	1.245	2.932	** 0.003**	**0.009**	1.738	1.036	2.918	**0.036**

*Bold value indicates *p* < 0.005. FDR-adjusted *p*-value < 0.05 was considered statistically significant. OS, overall survival; HR, hazard ratio; FDR, false discovery rate.*

Subsequently, in order to better understand the biological function of LINC02257 in colon adenocarcinoma, GO term and KEGG pathway analyses were performed after the normalization and preparation of transcriptome data from TCGA database (FDR < 0.050, *p*-value < 0.050, Figures 3C,D). As shown in Figure 3C, the biological processes and molecular functions strongly associated with LINC02257 expression were extracellular structure organization, ossification, collagen fibril organization, and connective tissue development. KEGG pathway analysis showed five pathways that had a significant correlation with LINC02257 expression: PI3K-Akt signaling pathway, Focal adhesion, Phagosome, Regulation of actin cytoskeleton, and MAPK signaling pathway (Figure 3D). Accordingly, these results suggest that LINC02257 expression may regulate extracellular structure organization through the PI3K-Akt signaling pathway, which is critically important in colon adenocarcinoma patients.

### LINC02257 Gene Expression Correlates With a Multi-Omics Analysis of 33 Cancer Types

#### Clinical Characteristics and Survival Analysis

**FIGURE 4:**
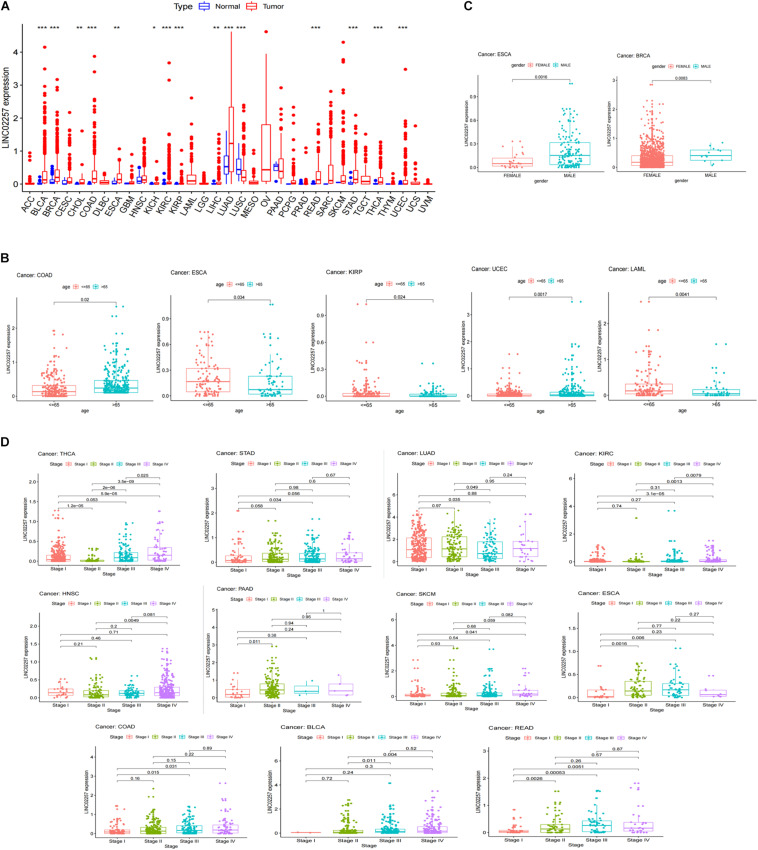
LINC02257 expression in 33 cancer types and its correlation with various clinicopathology features **(A)** LINC02257 in 33 tumor tissues and normal tissues; (*P < 0.001, *P < 0.01, *P < 0.05, no *P > 0.05) **(B)** In terms of age, COAD, ESCA, KIRP, UCEC, LAML show significant differences with LINC02257 expression. **(C)** As for gender, LINC02257 expression significantly correlates with ESCA and BRCA. **(D)** The stage of THCA, STAD, SKCM, READ, PAAD, LUAD, KIRC, HNSC, ESCA, COAD, BLCAexerts significant correlations with LINC02257 expression (*p* < 0.05 was considered significant).

**FIGURE 5:**
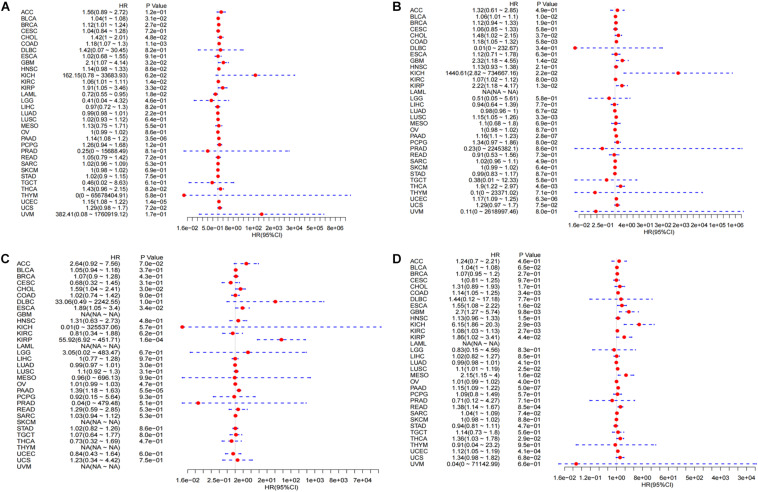
Integrated survival analysis of LINC02257 expression in 33 cancer types **(A)** The expression of LINC02257 is significant related to OS in BLCA, BRCA, CHOL, COAD, GBM, KIRC, LAML, LUAD, PAAD, UCEC. **(B)** In respect to DSS, BLCA, CHOL, COAD, GBM, KICH, KIRC, KIRP, LUSC, PAAD, THCA, UCEC show significant correlation to LINC02257. **(C)** As for DFI, CHOL, ESCA, KIRP, PAAD is significantly related to LINC02257 expression. **(D)** PFI of COAD, GBM, KICH, KIRC, KIRP, LUSC, MESO, PAAD, READ, THCA, UCEC is significantly related to LINC02257 expression. *P* < 0.05 indicates that the expression of LINC02257 is significantly correlated with the prognosis and survival of the patients. HR > 1, with the increase of LINC02257 expression, the risk of poor prognosis is higher).

A swarmplot in Figure 4A shows the difference in LINC02257 expression across 33 cancer types and their corresponding normal tissues from TCGA project. Consequently, the expression differences could be found in BLCA, BRCA, CHOL, COAD, ESCA, KICH, KIRC, KIRP, LIHC, LUAD, LUSC, READ, STAD, THCA, and UCEC. Further analysis was performed concerning the correlation of LINC02257 expression and clinicopathological features in patients with different types of cancer. In respect to age, COAD, ESCA, KIRP, UCEC, and LAML showed significant differences with LINC02257 expression (Figure 4B). Regarding gender, LINC02257 expression significantly correlated with ESCA and BRCA in Figure 4C. In addition, the stage of THCA, STAD, SKCM, READ, PAAD, LUAD, KIRC, HNSC, ESCA, COAD, and BLCA exerted significant correlations with LINC02257 expression, as shown in Figure 4D. In terms of survival analysis, the relationship of LINC02257 expression with OS, DSS, DFI, and PFI in cancers clarified that LINC02257 expression, acting as a high hazardous factor, was associated with the prognosis of multiple cancer types (Figure 5).

#### Multi-Omics Immunotherapy-Related Analysis

##### Tumor mutational burden

**FIGURE 6:**
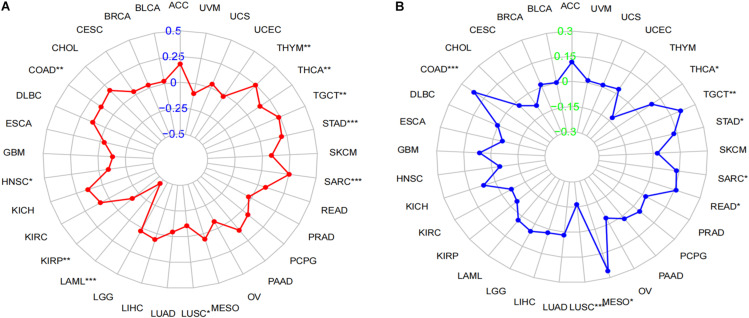
Correlation of LINC02257 expression with TMB and MSI in multiple cancer. **(A)** Correlation between TMB and LINC02257 expression. **(B)** Correlation between MSI and LINC02257 expression. Spearman’s correlation coefficients are shown above the bar graphs. (Spearman Correlation test, *p <* 0.05 was considered significant, *p < 0.05, **p < 0.0l, ***p < 0.001).

Tumor mutational burden is considered as a hallmark of genomic alterations, the increase of which could induce new antigens to facilitate immune recognition, producing better immunotherapy responses or favorable prognosis (Sabari et al., 2018). However, it is still unclear with regard to the significance of LINC02257 expression in TMB. TMB data were obtained from 33 cancers, followed by Spearman correlation analysis to assess the association of LINC02257 expression and TMB (Table 3, as a radar legend in Figure 6A). The result showed that LINC02257 expressions in COAD, HNSC, KIRP, LAML, LUSC, SARC, STAD, TGCT, and THCA were of great significance with TMB, among which LAML, SARC, and STAD exhibited the strongest correlation with TMB (*p* < 0.001).

**TABLE 3:** Correlation analysis regarding the association of LINC02257 expression and TMB.

**Cancer type**	**cor**	***p*-value**	**Cancer type**	**cor**	***p*-value**
ACC	0.18	0.113	LUSC	–0.10	0.022*
BLCA	0.02	0.639	MESO	0.05	0.631
BRCA	0.03	0.354	OV	–0.07	0.226
CESC	0.03	0.577	PAAD	0.12	0.134
CHOL	0.18	0.300	PCPG	0.062	0.378
COAD	0.13	0.008**	PRAD	–0.03	0.536
DLBC	0.12	0.463	READ	0.073	0.409
ESCA	–0.04	0.620	SARC	0.25	8.81E-05***
GBM	–0.14	0.099	SKCM	0.08	0.069
HNSC	–0.09	0.045*	STAD	0.20	9.28E-05***
KICH	0.14	0.274	TGCT	0.24	0.004**
KIRC	0.09	0.117	THCA	0.14	0.001**
KIRP	–0.17	0.004**	THYM	0.25	0.007**
LAML	–0.46	0.0001***	UCEC	–0.02	0.608
LGG	0.031	0.487	UCS	0.04	0.770
LIHC	0.059	0.266	UVM	–0.10	0.388
LUAD	–0.04	0.318			

***p < 0.001, **p < 0.01, *p < 0.05, no *p > 0.05. TMB, tumor mutational burden.

##### Microsatellite instability

MSI caused by hypermutability (gain or loss) of nucleotides from DNA elements, which was initially related to CRCs (Oki et al., 1999), is recently defined as a diagnostic hallmark of diverse cancer types and a promising marker for immune-checkpoint blockade therapy (Hause et al., 2016). However, there is no relevant research on MSI and LINC02257 expression. Accordingly, our subsequent experiment explored their relationship across multiple cancer types. To be more specific, after the MSI data were collected from TCGA database, Spearman analysis was performed between MSI and LINC02257 expression (Table 4, as a radar legend in Figure 6B). Results indicate that LINC02257 expressions in COAD, LUSC, MESO, READ, SARC, STAD, TGCT, and THCA were significantly related to MSI. Furthermore, LINC02257 expressions in COAD and LUSC had the most connection with MSI (*p* < 0.001).

**TABLE 4:** Correlation analysis regarding the association of LINC02257 expression and MSI.

**Cancer type**	**cor**	**p-value**	**Cancer type**	**cor**	**p-value**
ACC	0.12	0.308	LUSC	0.17	0.0002***
BLCA	0.001	0.976	MESO	0.26	0.019*
BRCA	0.01	0.642	OV	0.04	0.519
CESC	0.09	0.133	PAAD	0.02	0.806
CHOL	0.03	0.870	PCPG	0.05	0.535
COAD	0.21	7.175E-06***	PRAD	0.02	0.644
DLBC	0.002	0.989	READ	0.16	0.049*
ESCA	0.05	0.496	SARC	0.13	0.034*
GBM	0.062	0.452	SKCM	0.02	0.605
HNSC	0.05	0.306	STAD	0.13	0.011*
KICH	0.07	0.597	TGCT	0.21	0.009**
KIRC	0.06	0.245	THCA	0.09	0.042*
KIRP	0.05	0.431	THYM	0.13	0.172
LAML	0.03	0.805	UCEC	0.03	0.493
LGG	0.05	0.271	UCS	0.01	0.932
LIHC	0.02	0.676	UVM	0.01	0.908
LUAD	0.02	0.677			

***p < 0.001, **p < 0.01, *p < 0.05, no *p > 0.05. MSI, microsatellite instability.

##### Tumor microenvironment

**FIGURE 7:**
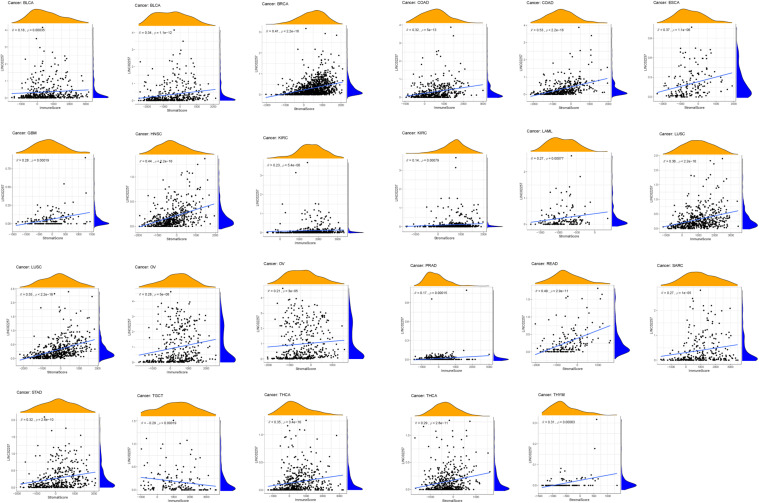
The relationship of LINC02257 expression in cancers and stromal and immune cells scores.

**FIGURE 8:**
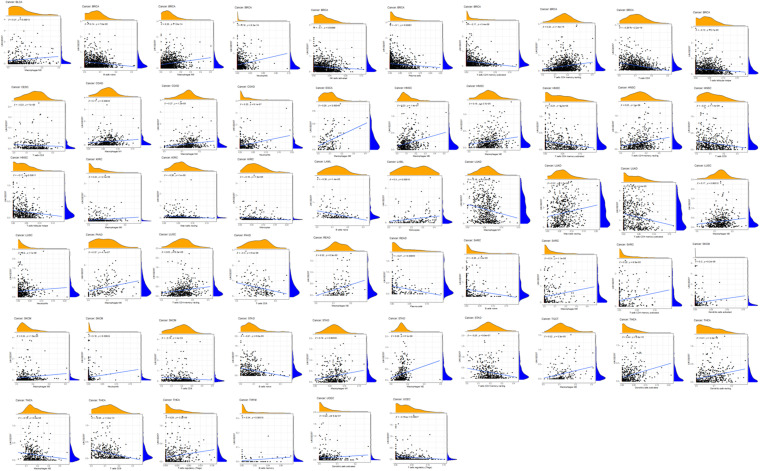
The expression of LINC02257 and the infiltrations of 22 immune cells in 33 different types of tumors (the type and content of immune cells on the *X*-axis, the gene expression distribution on the *Y*-axis; *P*-value < 0.001 and *R* > 0, indicates positive correlation and the existence of statistically significance difference).

Tumor microenvironment consists of tumor cells and non-tumor components. Stromal and immune cells are two main types of the later ones, the evaluation of which has been identified to be beneficial for cancer-targeted immunotherapies. An algorithm, called ESTIMATE, can help infer the fraction of stromal and immune cells and predict tumor purity in tumor samples. Hence, our study further analyzed the relationship of LINC02257 expression in cancers with stromal and immune cell scores calculated by ESTIMATE (Table 5 and Figure 7). A higher corresponding cell score might indicate a higher ratio of corresponding components in TME and lower corresponding tumor purity. Besides, the relationship between LINC02257 expression of LINC02257 and the infiltration of 22 immune cells was investigated in 33 different types of tumors, including CD8 + T cells, T cells (general), B cells, monocytes, TAMs, M1 and M2 macrophages, neutrophils, natural killer (NK) cells, and dendritic cells (DCs) (Figure 8). After the correlation adjustment by purity, the results revealed that LINC02257 expression was significantly correlated with the infiltration of various immune cells and different T cells. Furthermore, it was significantly related to macrophage infiltration in BLCA, BRCA, COAD, ESCA, HNSC, KIRC, LAML, LUAD, PAAD, READ, SARC, SKCM, STAD, TGCT, and THCA. Moreover, LINC02257 expression showed a significant association with T cell infiltration in BRCA, CESC, HNSC, LUSC, PAAD, SARC, SKCM, STAD, THCA, and UCEC.

**TABLE 5:** The relationship of LINC02257 expression in cancers and stromal and immune cells scores by ESTIMATE.

**Cancer type**	**Gene**	**Stromal score**	**Immune score**	**Cancer type**	**Gene**	**Stromal score**	**Immune score**
ACC	LINC02257	0.684	0.602	LUSC	LINC02257	2.07E−41	4.365E−17
BLCA	LINC02257	1.12E−12	0.0003	MESO	LINC02257	0.589	0.213
BRCA	LINC02257	1.87E−46	0.419	OV	LINC02257	2.97E−05	4.982E−08
CESC	LINC02257	0.002	0.694	PAAD	LINC02257	0.015	0.700
CHOL	LINC02257	0.407	0.480	PCPG	LINC02257	0.631	0.019
COAD	LINC02257	2.68E−35	4.98E−13	PRAD	LINC02257	0.002	0.0001
DLBC	LINC02257	0.120	0.540	READ	LINC02257	2.94E−11	0.006
ESCA	LINC02257	1.14E−06	0.199	SARC	LINC02257	0.001	1.00E−05
GBM	LINC02257	0.0001	0.004	SKCM	LINC02257	0.503	0.858
HNSC	LINC02257	8.73E−26	0.521	STAD	LINC02257	2.649E−10	0.008
KICH	LINC02257	0.374	0.104	TGCT	LINC02257	0.599	0.0001
KIRC	LINC02257	0.0008	5.43E−08	THCA	LINC02257	2.84E−11	3.39E−16
KIRP	LINC02257	0.101	0.197	THYM	LINC02257	0.0006	0.898
LAML	LINC02257	0.0008	0.054	UCEC	LINC02257	0.961	0.716
LGG	LINC02257	0.369	0.061	UCS	LINC02257	0.907	0.247
LIHC	LINC02257	0.005	0.022	UVM	LINC02257	0.974	0.937
LUAD	LINC02257	0.101	0.689				

##### Co-expression of LINC02257 and potential signaling pathways across different cancer types

**FIGURE 9:**
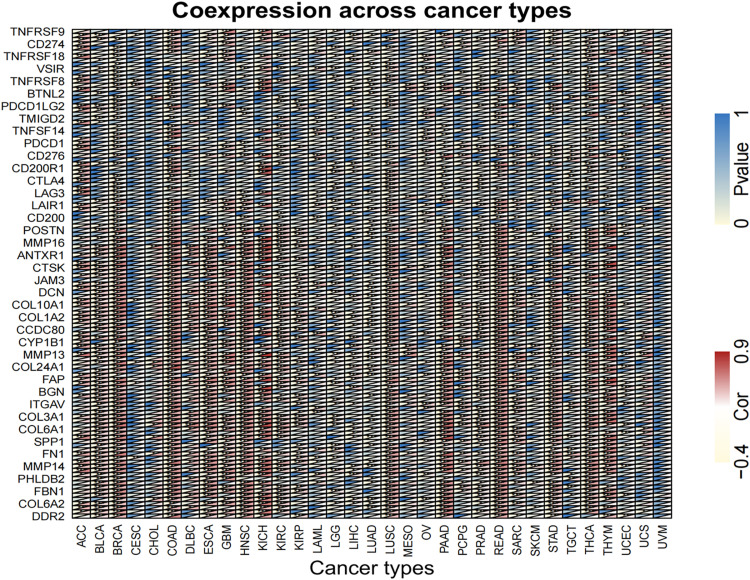
Analysis of the co-expression of LINC02257 in 33 tumors and its correlation (The deeper the blue, the higher the P-value and the lower the correlation. Yellow represents a negative correlation and red represents a positive correlation. White indicates no correlation. ***P < 0.001, **P < 0.01, *P < 0.05, no *P > 0.05).

Equally important, the co-expression of the target gene LINC02257 was analyzed in each of the 33 tumors and its correlation, gene correlation p-value uploaded in a supplementary material pdf file, named geneCor. pvalue.pdf, as illustrated in a heatmap (Figure 9). Finally, GSEA of potential function/pathway characteristics of target gene LINC02257 affecting the development of 33 tumors is elaborated in the Supplementary Material, which completes our integrated multi-omics study of LINC02257 expression in pan-cancers.

## Discussion

As a subclass of lncRNAs, eRNAs may regulate the expression of corresponding genes at the transcription level and hence be involved in the development of various types of cancer. Studies have revealed that there has been uncovered multiple information about colon cancer biomarkers and signatures, which are involved in a series of physio-pathological process (de la Chapelle and Hampel, 2010; Harada and Morlote, 2020). However, few studies have investigated the importance and mechanisms of eRNAs in colon cancer and other tumorigenesis. Meanwhile, current attention has been paid to a comprehensive analysis of immunotherapy-related studies regarding eRNA (Gu et al., 2019). Thus, our study provides insights into understanding the potential role of LINC02257 in tumor immunology and its use as a biomarker for cancer development.

In our study, eRNAs of prognostic value were identified for colon cancer. A total of 1,580 eRNAs were successfully obtained from 521 colon cancer samples, of which LINC02257 was found to have the most significant impact on patients’ survival with its corresponding target gene DUSP10, making it our top key eRNA for colon cancer. It has been documented that DUSP10 had a higher expression in colon cancer tissue than normal tissues and was involved in regulating colorectal tumorigeneses (Png et al., 2016), which was in correspondence with our results. Data from TCGA were further utilized to assess the relationship of LINC02257 with clinicopathological variables of colon cancer and survival analysis, which suggested that age and unregulated expression of LINC02257 could act as an independent unfavorable prognostic factor. Further, high LINC02257 expression might be associated with a more advanced tumor status and stage. Taken together, colon adenocarcinoma patients with high expression of LINC02257 are more susceptible to unfavorable prognosis than those with low expression, with the discovery of correlation with various clinical characteristics as well. Importantly, GO term analysis revealed that high LINC02257 expression could be connected with extracellular structure organization, ossification, collagen fibril organization, and connective tissue development. Meanwhile, KEGG pathway analysis showed five pathways that had a significant correlation with LINC02257 expression: PI3K-Akt signaling pathway, Focal adhesion, Phagosome, Regulation of actin cytoskeleton, and MAPK signaling pathway in colon cancer. Our study may pave the way to understanding that overexpression of LINC02257 in colon adenocarcinoma patients may alter PI3K-Akt signaling pathway, with its cascade involved in extracellular structure organization and ossification, providing potential targets for further investigation. As it has been demonstrated in the past, the PI3K-Akt signaling pathway is implicated in various cancer types through regulating cell proliferation and survival, including colon cancer, which is also in accordance with our analysis (Pal and Mandal, 2012).

It is still an unanswered question whether LINC02257, a functionally unannotated transcript, plays any roles in other cancer development. In our study, a comprehensive pan-cancer analysis was performed to clarify its aberrant expression with patients’ clinical characteristics and survival analysis (OS, DSS, DFI, and PFI) across 33 cancer types. The differential expression of LINC02257 between cancer and normal tissues was observed in many types of cancers, and LINC02257 is mainly more expressed in cancers, which means LINC02257 may function as an oncogene in tumorigenesis. It has been reported that age and gender may influence the prognosis of patients with colon adenocarcinoma, esophageal cancer, and other tumorigeneses (Jin et al., 2020; Badic et al., 2021; Chen et al., 2021). Based on this, we included these two factors in our study. Intriguingly, in addition to colon adenocarcinoma, expressions of LINC02257 in various tumor types, such as THCA and STAD, were linked with age, gender, and stage status. Concerning our survival analysis, LINC02257 expression could also play a detrimental role in several malignant tumors, especially in COAD and PAAD, which is consistent with the results of previous studies. Our analysis suggested a correlation between high LINC02257 and poor OS, DSS, and PFI. Besides, both age and LINC02257 expression are independent prognostic factors in colon adenocarcinoma. Then, we wanted to figure out the relationship concerning LINC02257, age, and survival. R package “mediation” (version 3.5.3) was performed for mediation analysis for age, as mediation between LINC02257 and survival showed non-significant average causal mediation effects (ACMEs; *p*-value = 0.79) and significant average direct effects (ADEs; *p*-value < 2E-16), which means that LINC02257 showed a direct and significant effect on survival and age, which was not a mediation value between LINC02257 and survival. The result pdf file, “mediation,” was uploaded in the Supplementary Material. Still, it inspires us to extend our understanding of LINC02257 in pan-cancer analysis, in both diagnosis and prognosis evaluations. In a recent study, TMB, TME, and MSI were correlated with immunotherapy (Goodman et al., 2017). Considering that colon cancer strongly links with MSI, TME, and other immunotherapy-related elements, it requires further in-depth understanding of the correlation between LINC02257 expression and immunotherapy-related analysis in cancer. As for TMB, our study discovered that LINC02257 expression in LAML, SARC, and STAD exhibited the strongest correlation with TMB, suggesting that LINC02257 expression in these three cancers is much likely to induce mutation-driven tumorigenesis and may provide implications for guidance on related drug therapy. Theoretically, patients with a higher TMB are warranted to get an ideal immunotherapy efficacy. When it comes to the TME, it is the environment where the tumor cells live, in which immune cells, endothelial cells, fibroblasts, and extracellular matrix are also located (Bolouri, 2015; Kurebayashi et al., 2018). The interaction among non-tumor components in TME, such as stromal and immune cells, can crucially influence the gene expression and the consequent clinical outcomes. In our study, the stromal and immune scores were obtained by the ESTIMATE algorithm to delineate their association with LINC02257 expression (Turley et al., 2015). Corresponding analysis revealed that LINC02257 expression was significantly associated with immune scores of BLCA, COAD, KIRC, LUSC, OV, PRAD, SARC, TGCT, and THCA, while stromal scores of BLCA, BRCA, COAD, ESCA, GBM, HNSC, KIRC, LAML, LUSC, OV, READ, STAD, THCA, and THYM were linked with the same gene expression. Accordingly, LINC02257 expression signature may be used to infer the ratio of the stromal and immune cells and thus predict the tumor purity in the TME. Besides, our study performed CIRBERSORT analysis to uncover the relationship between LINC02257 expression and immune cell infiltration in cancers. It was observed that LINC02257 expression was significantly associated with macrophages, B cells, neutrophils, NK cells, and T cells. Although we have yet to establish a cause–result relationship here, aberrant LINC02257 expression may alter tumor immune microenvironment across different cancer types. It in turn expands the pivotal role of eRNAs in the regulation of immune cell infiltration in various cancers. Apart from this, our study also elucidated the co-expression genes of LINC02257 and GSEA across different cancers. Concerning GSEA of LINC02257 expression across different cancer types, we uncovered that LINC02257 expression exerted shared mechanisms in that LINC02257 expression could regulate cell differentiation in BLCA, PRAD, SARC, TGCT, PADD, and LGG and hinder cell cycle (G1–S transition, sister chromatic segregation) in CESE, COAD, UCS, SKCM, and KICH, all of which are common carcinogenic patterns (Xiao et al., 2019; Garcia-Olivares et al., 2021; Tachiwana and Saitoh, 2021). Meanwhile, LINC02257 expression was also associated with NF-kappa B signaling pathway in LGG, providing us with various functional mechanisms of LINC02257 expression.

The notable strength of our study lies in the comprehensive omics analysis of eRNAs in 33 cancers, with the expectation to provide novel and robust evidence for potential cancer immunotherapy. And aberrant eRNAs expression may correlate with patient’s prognosis, especially that LINC02257 is an independent prognostic factor in COAD patients, which suggests that our study has potential clinical use in cancer prognosis assessment and future follow-up investigation. While it is currently unclear that the precise mechanism is in regard to LINC02257 involved in COAD tumorigenesis, our study shows that the PI3K-Akt signaling pathway could possibly promote COAD occurrence and progression. However, several limitations existed in our study. Firstly, the newly identified LINC02257 we uncovered across cancers warrants basic experimental validation, including the potential regulatory mechanisms. Secondly, our study explored the prognostic value of LINC02257 in multiple cancers via different ways (OS, DSS, PFI, and DFI), which, however, is still insufficient for our understanding and mechanism explanation. Thirdly, although we evaluated TMB, MSI, TME, and other immunotherapy-related factors, we did not analyze the association between LINC02257 expression and common immune checkpoint genes, such as CD276 and CD200, which could be potential indicators of patients’ immunotherapy response. So the predictive value of LINC02257 regarding the immunotherapy response remains to be well-documented in the future.

To conclude, our study for the first time unveils key eRNA, LINC02257, as a new biomarker for colon and other cancers and attempts to elucidate its different roles in clinical parameters and prognosis. With a comprehensive analysis, LINC02257 may serve as a potential tool to enhance our understanding of the diagnosis and prognosis prediction of cancers and may be a promising immune-related therapeutic target for the precision treatment of malignant diseases in the future.

## Data Availability Statement

The original contributions presented in the study are included in the article/Supplementary Material, further inquiries can be directed to the corresponding author/s.

## Ethics Statement

As this work benefited from the database of TCGA, informed consent was not applicable. This study was approved by the Xiangya Hospital, Central South University Ethics Committee, and the usage of the information and specimens collected has been handled and anonymized conformed to the ethical and legal standards.

## Author Contributions

JX wrote the article. YL and JY helped in the preparation of the manuscript, construction of tables and citation of references, and the critical revision of the literature. All authors contributed to the article and approved the submitted version.

## Conflict of Interest

The authors declare that the research was conducted in the absence of any commercial or financial relationships that could be construed as a potential conflict of interest.

## Footnotes

1 https://portal.gdc.cancer.gov/

2 http://cancergenome.nih.gov/abouttcga/policies/publicationguidelines

3 http://cibersort.stanford.edu/

## Abbreviations

ACC: adrenocortical carcinoma
AR: androgen receptor
BLCA: bladder urothelial carcinoma
BRCA: breast invasive carcinoma
CESC: cervical squamous cell carcinoma and endocervical adenocarcinoma
CHOL: cholangiocarcinoma
COAD: Colon adenocarcinoma
CRC: colorectal cancer
DFI: disease-free interval
DLBC: lymphoid neoplasm diffuse large B-cell lymphoma
DSS: disease-specific survival
eRNA: enhancer RNA
ESCA: esophageal carcinoma
ESTIMATE: Estimation of stromal and Immune cells in Malignant Tumors using Expression data
FDR: false discovery rate
GBM: glioblastoma multiforme
HNSC: head and neck squamous cell carcinoma
KICH: kidney chromophobe
KIRC: kidney renal clear cell carcinoma
KIRP: kidney renal papillary cell carcinoma
KLK3e: Kallikrein-related peptidase 3 eRNA
LAML: acute myeloid leukemia
LGG: brain lower-grade glioma
LIHC: liver hepatocellular carcinoma
LUAD: lung adenocarcinoma
LUSC: lung squamous cell carcinoma
MESO: mesothelioma
MSI: microsatellite instability
ncRNA: non-coding RNA
OS: overall survival
OV: ovarian serous cystadenocarcinoma
PAAD: pancreatic adenocarcinoma
PCPG: pheochromocytoma and paraganglioma
PFI: progression-free interval
PRAD: prostate adenocarcinoma
READ: rectum adenocarcinoma
RNAP II: RNA polymerase II
SARC: sarcoma
SKCM: skin cutaneous melanoma
STAD: stomach adenocarcinoma
TCGA: The Cancer Genome Atlas
TGCT: testicular germ cell tumors
THCA: thyroid carcinoma
THYM: thymoma
TMB: tumor mutational burden
TME: tumor microenvironment
UCEC: uterine corpus endometrial carcinoma
UCS: uterine carcinosarcoma
UVM: uveal melanoma
