# Supplementary material for: LINC02257, an Enhancer RNA of Prognostic Value in Colon Adenocarcinoma, Correlates With Multi-Omics Immunotherapy-Related Analysis in 33 Cancers
Source: Front Mol Biosci. 2021 Apr 30;8:646786. doi: 10.3389/fmolb.2021.646786 (PMC8121256; doi:10.3389/fmolb.2021.646786)
Supplement: Supplementary file 1 [file Table_1.DOCX]

| gene | KM | FDR |
| --- | --- | --- |
| LINC02257 | 0.00012532 | 0.03705623 |
| AC053527.2 | 0.00236009 | 0.23259758 |
| ELFN1-AS1 | 0.00372687 | 0.23259758 |
| AP003555.2 | 0.00450645 | 0.23259758 |
| AC022784.1 | 0.00500228 | 0.23259758 |
| ELFN1 | 0.00517534 | 0.23259758 |
| AP003555.1 | 0.00550634 | 0.23259758 |
| LINC02542 | 0.00710717 | 0.26269185 |
| LINC00649 | 0.00900198 | 0.26719712 |
| RRP7BP | 0.01000131 | 0.26719712 |
| AC092944.1 | 0.01159129 | 0.26719712 |
| MYOSLID | 0.01159681 | 0.26719712 |
| AC021028.1 | 0.01174722 | 0.26719712 |
| STEAP1B | 0.01517609 | 0.30114 |
| AFDN-DT | 0.01529553 | 0.30114 |
| AL683813.2 | 0.01629478 | 0.30114 |
| AC108047.1 | 0.01882269 | 0.32257592 |
| AC108751.4 | 0.02061273 | 0.32257592 |
| TMEM184A | 0.02072743 | 0.32257592 |
| LINC02062 | 0.0223703 | 0.33073632 |
| C2orf92 | 0.02682689 | 0.36068484 |
| CHST12 | 0.02691733 | 0.36068484 |
| ZNRF2P2 | 0.03039512 | 0.36068484 |
| MALINC1 | 0.03076446 | 0.36068484 |
| AC013287.1 | 0.03216766 | 0.36068484 |
| CUTALP | 0.03262386 | 0.36068484 |
| LINC00261 | 0.03300306 | 0.36068484 |
| AC046134.2 | 0.03415434 | 0.36068484 |
| AL136307.1 | 0.03708684 | 0.36575253 |
| LINC00174 | 0.03760549 | 0.36575253 |
| AL353747.3 | 0.03940884 | 0.36575253 |
| LINC00513 | 0.04001438 | 0.36575253 |
| AL031289.1 | 0.0408189 | 0.36575253 |
| MIR4435-2HG | 0.04207077 | 0.36588243 |
| AC139149.1 | 0.04345036 | 0.36678908 |
| AL139246.2 | 0.04486095 | 0.36678908 |
| APELA | 0.04674045 | 0.36678908 |
| AC012368.1 | 0.04831255 | 0.36678908 |
| LINC02381 | 0.04837723 | 0.36678908 |

***supplementary table 1 eRNA gene expressions associated with the prognosis of colon adenocarcinoma***

***(Kaplan–Meier log-rank test, p < 0.05, FDR-adjusted p-value<0.05 was considered significant )***.
