# Supplementary figures and images for: LINC02257, an Enhancer RNA of Prognostic Value in Colon Adenocarcinoma, Correlates With Multi-Omics Immunotherapy-Related Analysis in 33 Cancers
Source: Front Mol Biosci. 2021 Apr 30;8:646786. doi: 10.3389/fmolb.2021.646786 (PMC8121256; doi:10.3389/fmolb.2021.646786)

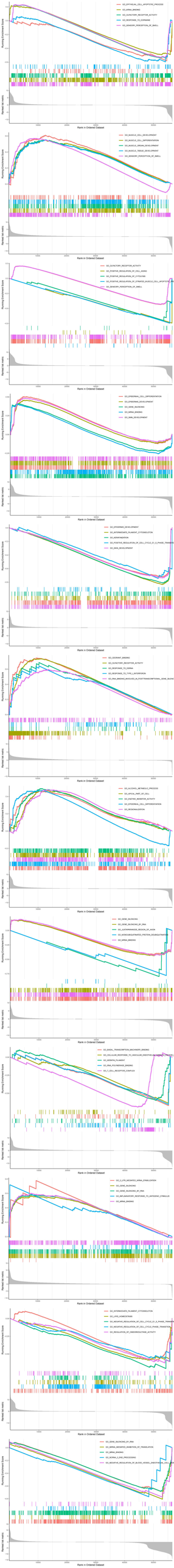

Supplement: Supplementary file 3 [file Image_1.JPEG]
